# Auditory Cortex Neurons Show Task-Related and Learning-Dependent Selectivity toward Sensory Input and Reward during the Learning Process of an Associative Memory Task

**DOI:** 10.1523/ENEURO.0046-22.2022

**Published:** 2022-05-27

**Authors:** Shogo Takamiya, Kazuki Shiotani, Tomoya Ohnuki, Yuma Osako, Yuta Tanisumi, Shoko Yuki, Hiroyuki Manabe, Junya Hirokawa, Yoshio Sakurai

**Affiliations:** 1 Laboratory of Neural Information, Graduate School of Brain Science, Doshisha University, Kyoto 610-0394, Japan; 2 Japan Society for the Promotion of Science, Tokyo 102-0083, Japan; 3 Laboratory of Brain Network Information, College of Life Sciences, Ritsumeikan University, Shiga 525-8577, Japan; 4 Department of Life Sciences, Graduate School of Arts and Sciences, The University of Tokyo, Tokyo 153-0041, Japan

**Keywords:** associative learning, auditory cortex, neural recordings, rats

## Abstract

The activity of primary auditory cortex (A1) neurons is modulated not only by sensory inputs but also by other task-related variables in associative learning. However, it is unclear how A1 neural activity changes dynamically in response to these variables during the learning process of associative memory tasks. Therefore, we developed an associative memory task using auditory stimuli in rats. In this task, rats were required to associate tone frequencies (high and low) with a choice of ports (right or left) to obtain a reward. The activity of A1 neurons in the rats during the learning process of the task was recorded. A1 neurons increased their firing rates either when the rats were presented with a high or low tone (frequency-selective cells) before they chose either the left or right port (choice-direction cells), or when they received a reward after choosing either the left or right port (reward-direction cells). Furthermore, the proportion of frequency-selective cells and reward-direction cells increased with task acquisition and reached the maximum level in the last stage of learning. These results suggest that A1 neurons have task- and learning-dependent selectivity toward sensory input and reward when auditory tones and behavioral responses are gradually associated during task training. This selective activity of A1 neurons may facilitate the formation of associations, leading to the consolidation of associative memory.

## Significance Statement

The activity of the primary auditory cortex (A1) neurons is modulated not only by sensory inputs but also by other task-related variables in associative learning. However, it is unclear how A1 neural activity changes dynamically in response to these variables during the learning processes of associative memory tasks. This study showed that neurons in A1 have the task-related and learning-dependent selectivity toward sensory inputs and rewards when auditory tones and behavioral responses are gradually associated during the training of an associative memory task. We conclude that this selective activity of A1 neurons might facilitate the formation of associations, leading to the consolidation of associative memory.

## Introduction

Associative learning between stimuli and behavioral responses induces modulation of neural activity in several brain regions. For successful associative learning, neural circuits integrate stimulus information with correct choices and rewards. This means that sensory regions, in addition to the hippocampal and motor regions, modulate their neural activity during learning processes (Weinberger, 2004). During learning with auditory stimuli, the primary auditory cortex (A1) neurons, each of which intrinsically responds to a specific tone frequency (tonotopic representation), become tuned to the frequency of the conditioned tone, and the proportion of these neurons increases with training (Bakin and Weinberger, 1990; Weinberger, 2004). Thus, A1 neurons have high plasticity, and their firing preference is modulated by auditory associative learning (Irvine, 2018).

The activity of A1 neurons is modulated not only by inputs, but also by other task-related variables (Caras and Sanes, 2017; Lin et al., 2019). Guo et al. (2019) reported that A1 neurons show choice-direction selectivity, whereas Bigelow et al. (2019) reported that motor movements modulate A1 neuronal activity. Furthermore, reward-related activity has been detected in A1 neurons (Brosch et al., 2011). Therefore, it is likely that A1 neurons represent important variables related to associative memories that form relations among auditory stimuli, behavioral choices, and rewards. However, it is unclear how A1 neural activity changes dynamically in response to these variables during the learning processes related to associative memory tasks. Hence, we developed an auditory associative memory task for rats (Takamiya et al., 2021), in which associative memory is gradually acquired over several days. In this task, the rats were required to associate tone frequencies (high and low) with the choice of ports (right and left) to obtain a reward. We recorded the firing activity of A1 neurons in the rats during the learning process of the task. We hypothesized that the number of task-related cells in A1 gradually increased; that is, their firing was gradually modulated during task acquisition.

## Materials and Methods

### Animals

Six male Wistar albino rats (Shimizu Laboratory Supplies) were individually housed and maintained on a laboratory light/dark cycle (lights on at 8:00 A.M. and off at 9:00 P.M.). The rats were placed under food restriction with *ad libitum* access to water. The animals were maintained at ∼80% of their baseline weight throughout the experiments. All experiments were conducted in accordance with the guidelines for the care and use of laboratory animals provided by the Animal Research Committee of Doshisha University.

### Apparatus

**Figure 1.**
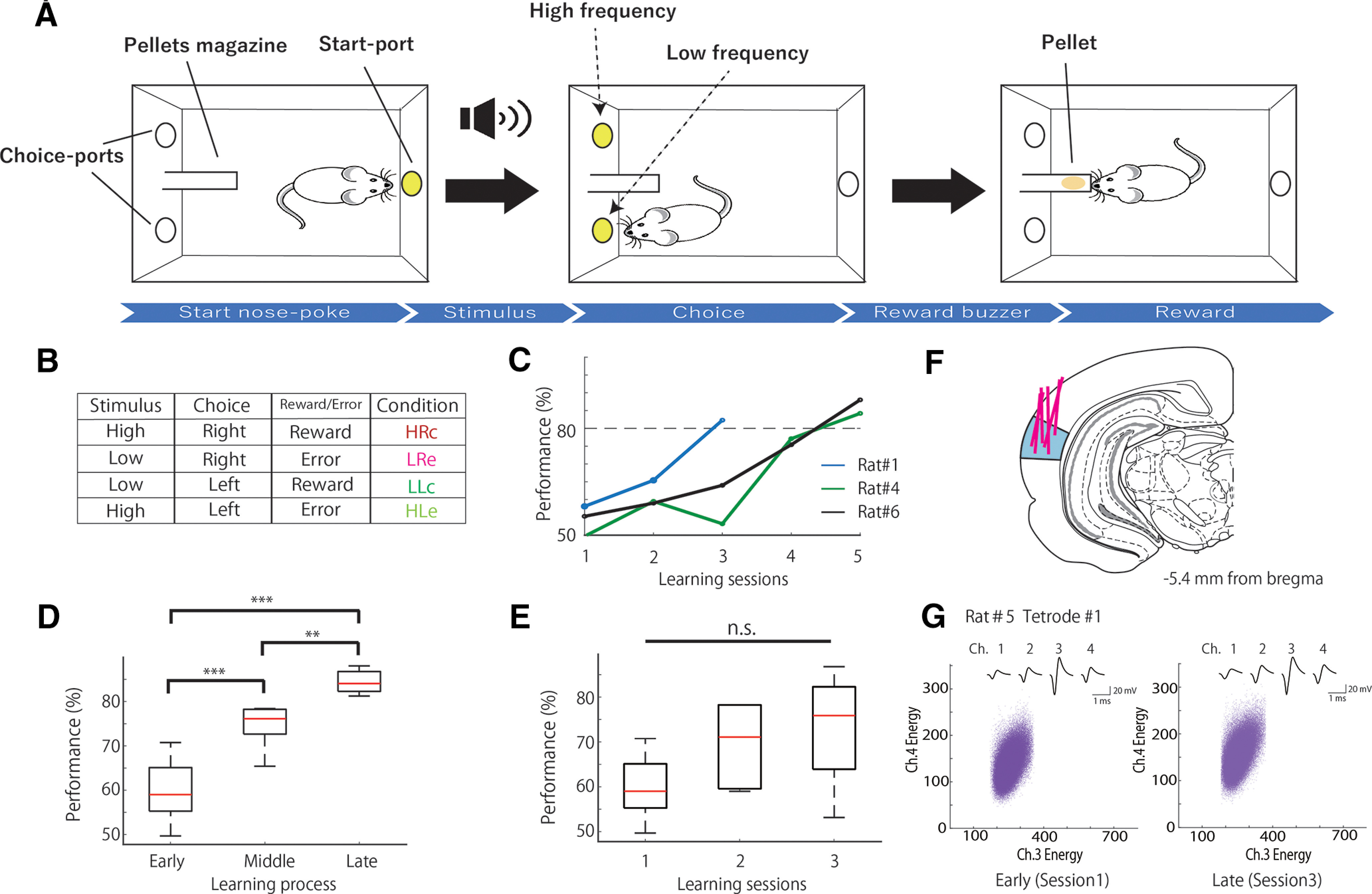
Associative memory task and behavioral performance. ***A***, Schematic representation of the task. ***B***, Table of task conditions, and combinations of stimuli, choices, and outcomes, with their respective acronyms. ***C***, Examples of performance in the associative memory task (rats 1, 4, and 6). ***D***, Mean number of correct performances in the task during each learning stage in all rats (*n* = 6). ***E***, Mean number of correct performances in the task in the first, second, and third sessions in all rats (*n* = 6). ***F***, Coronal section view showing the recording sites in the primary auditory cortex (blue). Red lines indicate the track of the movable tetrodes. ***G***, An example of spike waveform consistency during the early (left) and late (right) stages. Top, The averaged spike waveforms on each channel of tetrode 1. Bottom, Scatter plots of the spike clusters during the early (left) and late (right) stages. Each purple point represents a spike. The horizontal and vertical axes represent the energy of the spikes as detected by channels 3 and 4 of tetrode 1, respectively. ***p <* *0.01; **p <* *0.025; n.s.; not significant.

Behavioral training was performed in an operant chamber (23 × 11 × 35 cm; Ohara-Ika) with two ports in the front wall and a port in the back wall for a snout-poke response (Fig. 1*A*). The distance between the right and left ports was 70 mm. Each port was equipped with an LED light and an infrared sensor, which detected the nose-poke responses in the animal. A loudspeaker (15 cm in diameter) was placed 15 cm above the top of the chamber for the sound stimuli. A food dispenser delivered a 45 mg food pellet to a magazine located 1.5 cm above the floor and on the middle of the front wall. The chamber was enclosed in a soundproof box (Brain Science Idea). All the events were controlled using a personal computer (NEC).

### Behavioral task

#### Associative memory task

Six rats were trained in an associative memory task with tones, in which they were required to associate tone frequencies (high and low) and port locations (right and left; Fig. 1*A*). All animals learned the task with the same rule; that is, they learned a high tone-right reward association and a low tone-left reward association (Fig. 1*B*). At the start of each trial, the light in port in the back wall was turned on, and a high or low tone was randomly presented when the rat poked its snout into the port. Subsequently, the light of the port in the back wall was turned off, and the lights in the right and left ports in the front wall were turned on. When the tone was high, it was correct for the rat to poke its snout into the right port. When the tone was low, the correct response was to choose the port on the left. Immediately after the choice response, the light from the ports in the front wall were turned off. A food pellet was delivered into the pellet magazine along with a buzzer noise when the rat made the correct choice (Fig. 1*A*), and the light in port in the back wall was turned on again to start the next trial. When the rat chose incorrect ports, a timeout was imposed, and the lighting on the back wall port to start the next trial was delayed for 5 s. When the rat did not choose either the right or left port for 10 s, the trial was canceled. The selection of high versus low tones was pseudorandom, long runs of more than four successions of one type of tone were not permitted, and there was an equivalent number of high-tone and low-tone trials in each session.

Rats were trained with 3 and 1 kHz tone stimuli until their accuracy reached >80%. After completion of the training, the rats underwent surgery for electrode implantation. A week after surgery, the rats were trained in the same task, but the tone stimuli were 10 and 6 kHz. They were trained until the choice accuracy was >80%, and we recorded all neural activity during the training process. Each training session consisted of 150–250 trials/d.

#### Passive tone condition

Three of the six rats were used for the passive tone condition after training the associative memory task. The rats were randomly exposed to 3 s of 10 and 6 kHz tones presented 50 times each at 20 s intervals in the operant chamber.

### Surgery

The surgical procedures were almost identical to those reported in previous studies (Ohnuki et al., 2020; Osako et al., 2021; Takamiya et al., 2021). Rats were anesthetized with 2.5% isoflurane before surgery, which was maintained throughout the surgical procedure. We monitored the body temperature and depth of anesthesia of the rats as needed. An eye ointment was used to keep the eyes of the animals moistened throughout surgery. A craniotomy was performed over the right auditory cortex (distance relative to bregma: AP, −5.5 to −5.4 mm; ML, 6.7–7.2 mm; 1.0 mm below the brain surface), and custom-designed tetrodes attached to a microdrive were vertically implanted using a stereotactic manipulator. A stainless steel screw was placed over the cerebellum and served as the ground during the recording.

### Recording

For each rat, eight tetrodes composed of four tungsten wires (12.5 μm; California Fine Wire) were used for extracellular recordings. Each tetrode was covered by a polyimide tube (A-M Systems), and tetrodes were placed 100 μm apart. The tip impedance was 200–1000 kΩ at a frequency of 1 kHz. The signals were recorded using a head stage (Intan Technologies) and a multichannel electrophysiology acquisition board (Open Ephys) at a sampling rate of 30 kHz and bandpass filtered between 0.3 and 6 kHz. The mean activity of all the tetrodes was used as a reference. During a week of postsurgical recovery, the tetrodes were advanced by 20 μm/d until the firing of some cells was observed. We did not move the tetrodes during the training of the rats to record the same cell population throughout the training process.

### Histology

After the experiment, each rat was anesthetized with sodium pentobarbital and perfused with PBS and 4% paraformaldehyde. The brains were removed and postfixed in 4% paraformaldehyde, and 50 μm coronal sections of the brains were prepared to confirm the recording sites.

### Data analysis

Spike-sorting analysis was performed using MATLAB (MathWorks). To detect single-neuron activity, spikes were manually clustered with MClust (A.D. Redish; https://redishlab.umn.edu/mclust) in MATLAB. Only those neurons that met the following criteria were included for further analyses: (1) spikes with sufficient isolation quality (isolation distance ≥ 15); and (2) spikes with reliable refractory periods of 2 ms (violations were <1% for all spikes).

#### Detecting task-related neurons

**Figure 2.**
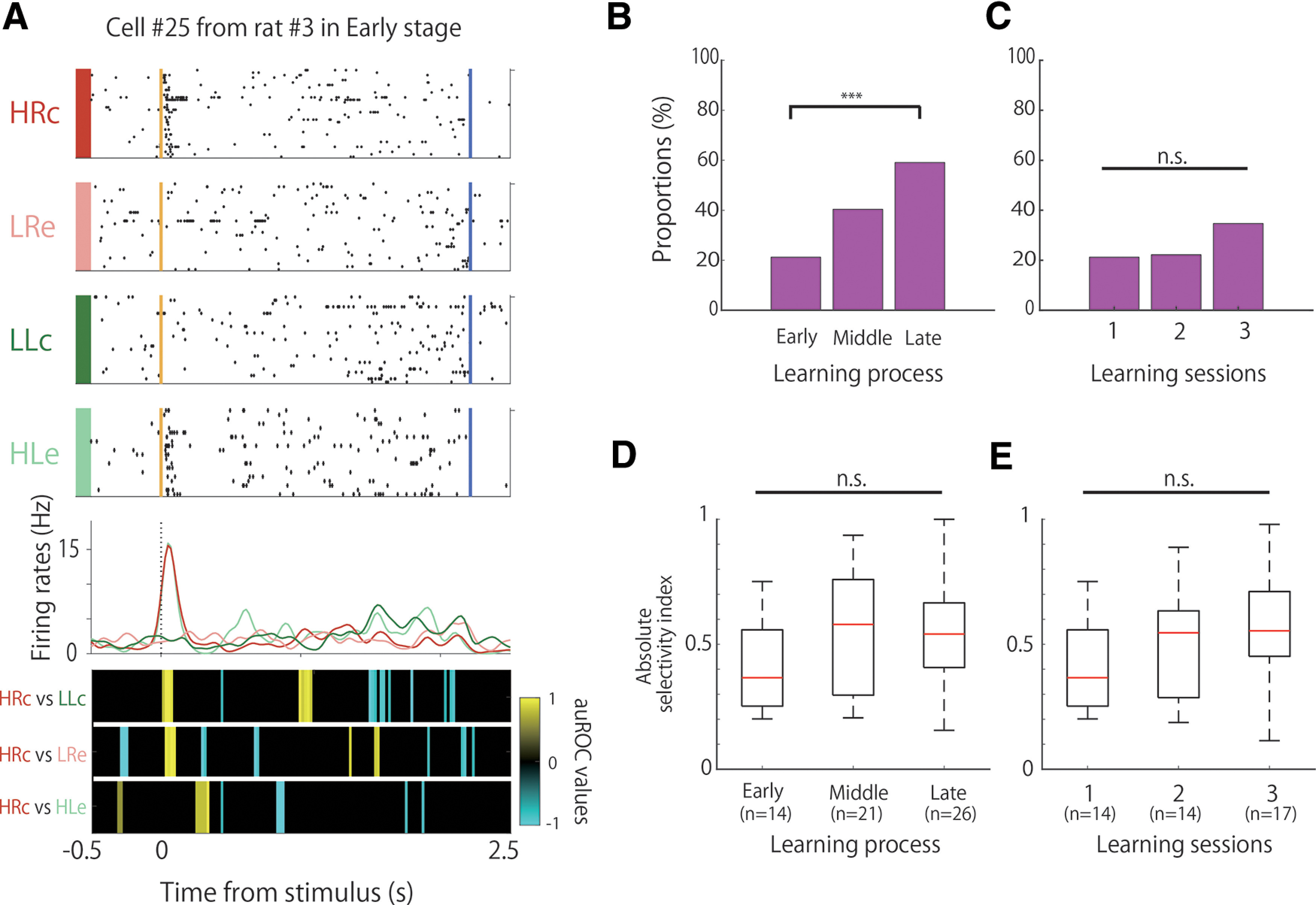
Proportion and selectivity of frequency-selective cells during learning. ***A***, An example of the firing patterns of the frequency-selective cells, which demonstrated firing modulation in high-tone trials. The data from 0.5 s before to 2.5 s after stimulus onset are shown. Top panels, Raster plots for each task condition; yellow and blue lines indicate the tone stimulus onset and choice response times, respectively. Middle panels, EASHs of HRc trials (red), LRe trials (pink), LLc trials (green), and HLe trials (light green). Dashed lines indicate the stimulus onset times. Bottom panels, auROC values of the task conditions for defining frequency-selective cells. The color scale represents the auROC values. Nonsignificant auROC values close to 0 are expressed in black. ***B***, Comparison of the proportion of frequency-selective cells among the learning stages. ***C***, Comparison of the proportion of frequency-selective cells among the learning sessions. ***D***, Comparison of the selectivity of frequency-selective cells among the learning stages. ***E***, Comparison of the selectivity of frequency-selective cells among the learning sessions. ***p <* *0.01; n.s.; not significant.

To evaluate the task-related neurons, we computed peristimulus time histograms using a 20 ms bin width and smoothed them by convolving spike trains with a 40-ms-wide Gaussian filter in the following four trial outcome conditions: high-right-correct (HRc), low-right-erroneous (LRe), low-left-correct (LLc), and high-left-erroneous (HLe; Fig. 1*B*). To examine the relationship between firing rate modulation and the development of behavioral epochs in the behavioral task, we used event-aligned spike histograms (EASHs). As the choice epoch durations varied for each trial, the median duration from the time when the stimulus was presented to the time when the rats chose either port was calculated, resulting in a median time of 2.38 s. The spike timing during choice epoch of each trial was linearly transformed into a median duration (Fig. 2*A*). For each neuron, we performed an area under the receiver operating characteristic curve (auROC) analysis (Green and Swets, 1966; Shiotani et al., 2020; Tanisumi et al., 2021). Starting from the baseline period of each condition (1 s of fixation before the start of the trial), the ROC value was calculated for each 100 ms bin. This bin was then stepped forward in 20 ms increments until 5 s after the start of the trial. We also performed auROC analysis to evaluate the selectivity of each neuron by comparing the firing rate of each trial condition (HRc vs LRe, HRc vs HLe, HRc vs LLc, LLc vs HLe, LLc vs LRe, and HLe vs LRe) in the same manner as described previously. We used permutation tests (1000 iterations) to determine the statistical significance (*p *<* *0.01). auROC values were defined as 2 × (auROC curve − 0.5), ranging from −1 to 1. The sample size for the test was no fewer than eight trials. This is sufficient because the minimal sample size used in a previous study was four trials (Felsen and Mainen, 2008).

We defined several task-related neurons under the following conditions: frequency-selective cells modulated their firing rates in response to tone differences (high or low). These cells consist of high-frequency-selective and low-frequency-selective cells. High-frequency-selective cells were defined as follows: (1) auROC values of HRc versus LLc that were significant for five bins in a row from the start of the trial to the points of choice; (2) the auROC values of HRc versus LRe that were significant for the same period as in 1; and (3) the auROC values of HRc versus HLe that were not significant for the same period as in 1 (Fig. 2*A*).

We defined low-frequency-selective cells as follows: (1) auROC values of LLc versus HRc that were significant for five bins in a row from the start of the trial to the points of choice; (2) auROC values of LLc versus HLe that were significant for the same period as in 1; and 3 auROC values of LLc versus LRe that were not significant for the same period as in 1.

#### Sound-evoked cells

**Figure 3.**
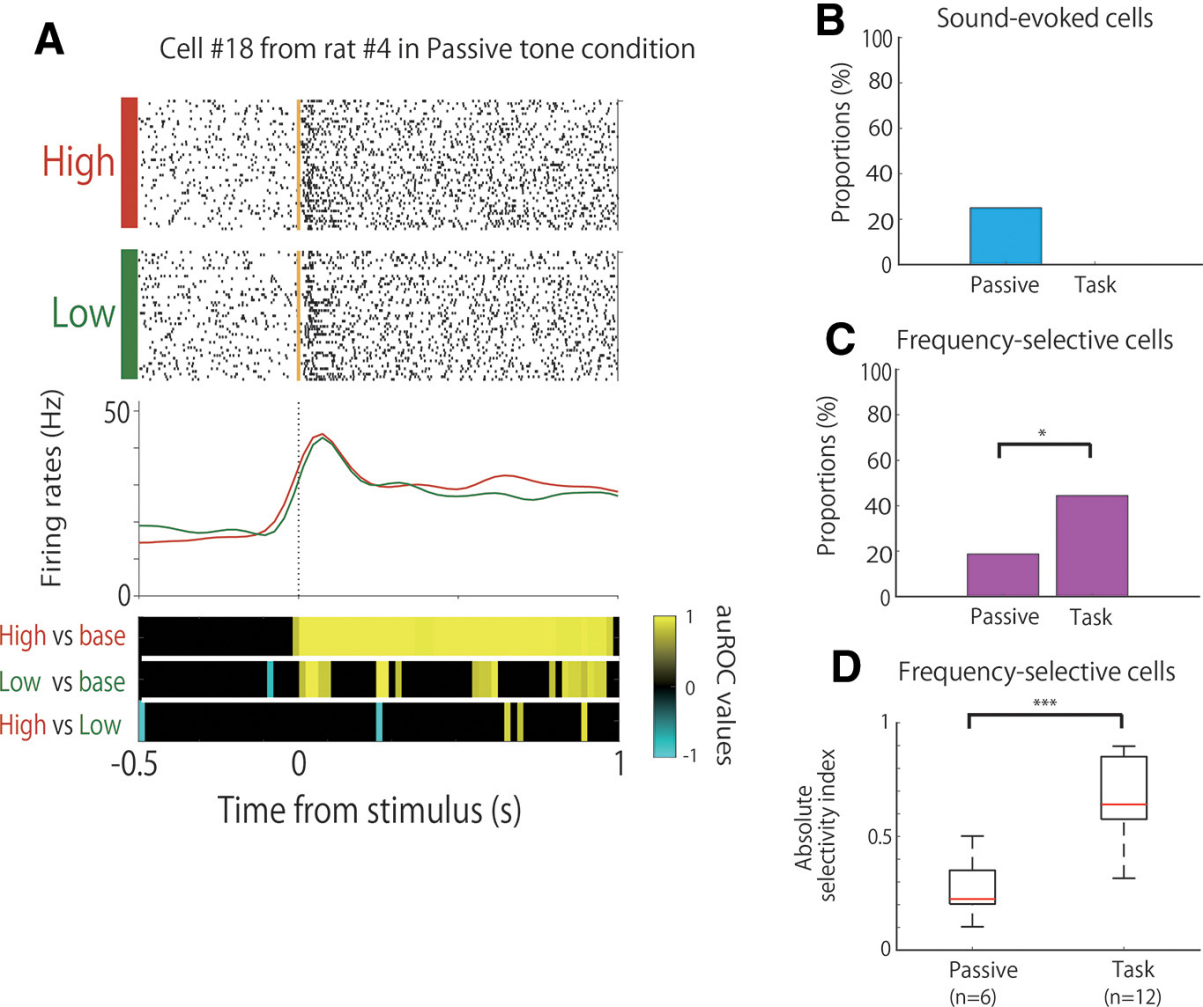
Comparison of the neural activity between the task and passive tone condition. ***A***, An example of the firing patterns from sound-evoked cells, which demonstrated firing increases during stimulus onset. The data from 0.5 s before to 1 s after stimulus onset are shown. Top panels, Raster plots for each tone condition. Yellow lines indicate the stimulus tone onset times. Middle panels, PSTHs of the high-tone trials (red) and low-tone trials (green). Dashed lines indicate the stimulus onset times. Bottom panels, auROC values of the task conditions for defining sound-evoked cells. The color scale represents the auROC values. Nonsignificant auROC values close to 0 are expressed in black. ***B***, Comparison of the proportion of sound-evoked cells between the task and passive tone condition. ***C***, Comparison of the proportion of frequency-selective cells between the task and passive tone condition. ***D***, Comparison of the selectivity of frequency-selective cells between the task and passive tone condition. ***p <* *0.01; *p,0.05; n.s.; not significant.

The auROC values of all conditions were calculated by comparing the firing rates of each period to the baseline of each condition that were significant for five bins in a row from the start of the trial up to 1 s after the response (Fig. 3*A*).

**Figure 4.**
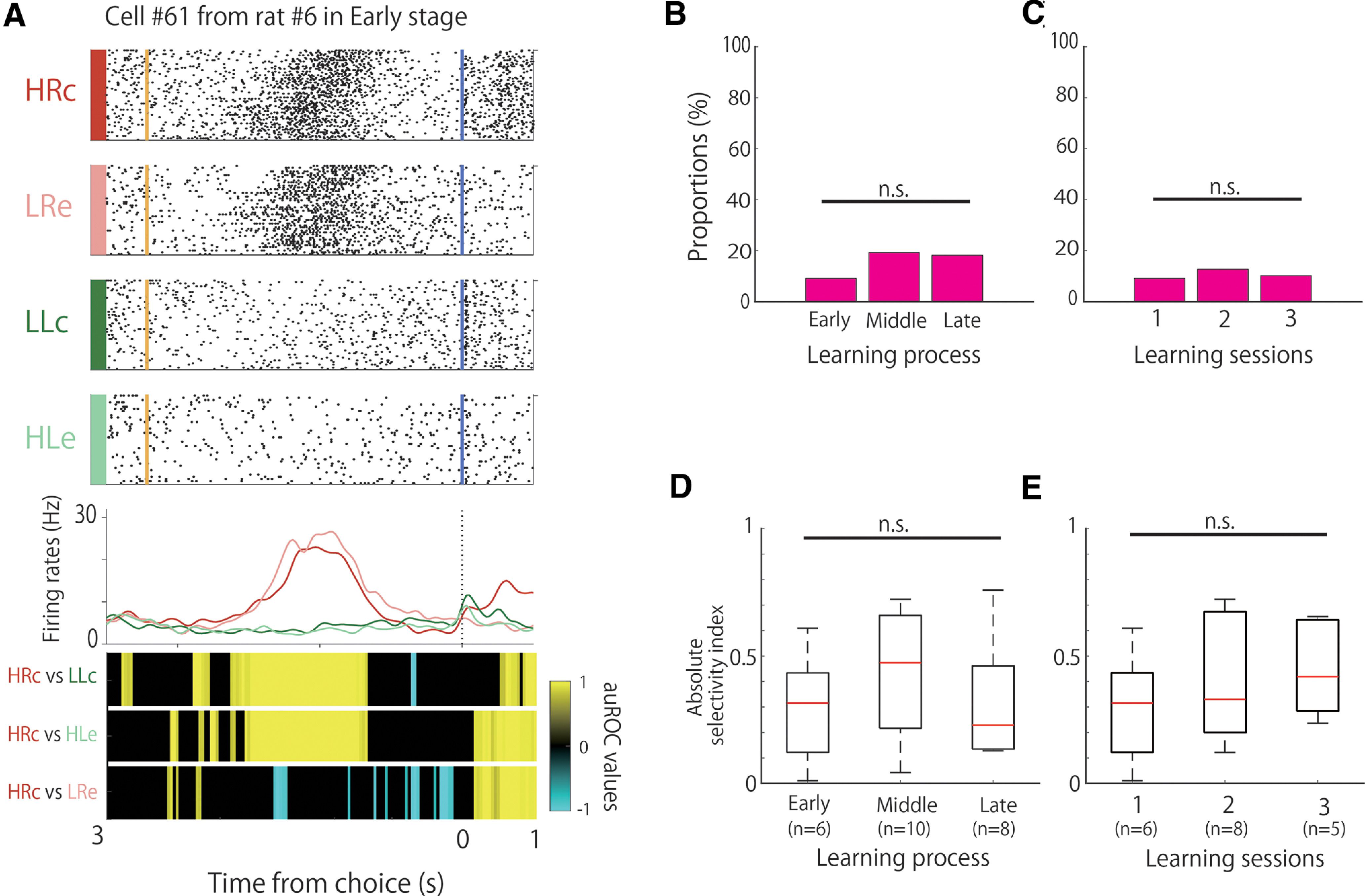
Proportion and selectivity of choice direction-selective cells during learning. ***A***, An example of the firing patterns of choice direction-selective cells, with firing increases in the right choice trials presented. The data from 3 s before to 1 s after the choice responses are shown. Top panels, Raster plots for each task condition; yellow and blue lines indicate the tone stimulus onset and choice response times, respectively. Middle panels, EASHs of each task condition. Dashed lines indicate the choice response times. Bottom panels, auROC values of the task conditions for defining choice direction-selective cells. The color scale represents the auROC values. Nonsignificant auROC values close to 0 are expressed in black. ***B***, Comparison of the proportion of choice direction-selective cells among the learning stages. ***C***, Comparison of the proportion of choice direction-selective cells among the learning sessions. ***D***, Comparison of the selectivity of choice direction-selective cells among the learning stages. ***E***, Comparison of the selectivity of choice direction-selective cells among the learning sessions. n.s.; not significant.

Choice-direction cells modulated their firing rates in response to the choice direction (right or left). These cells consisted of right choice-selective and left choice-selective cells. We defined right choice-selective cells as follows: (1) auROC values of HRc versus LLc that were significant for five bins in a row from the start of the trial to the points of choice; (2) auROC values of HRc versus HLe that were significant for the same period as in 1; and (3) auROC values of HRc versus LRe that were not significant for the same periods as in 1 (Fig. 4*A*).

We defined left choice-selective cells as follows: (1) auROC values of LLc versus HRc that were significant for five bins in a row from the start of the trial to the points of choice; (2) auROC values of LLc versus LRe that were significant for the same period as in 1; and (3) auROC values of LLc versus HLe that were not significant for the same period as in 1.

**Figure 5.**
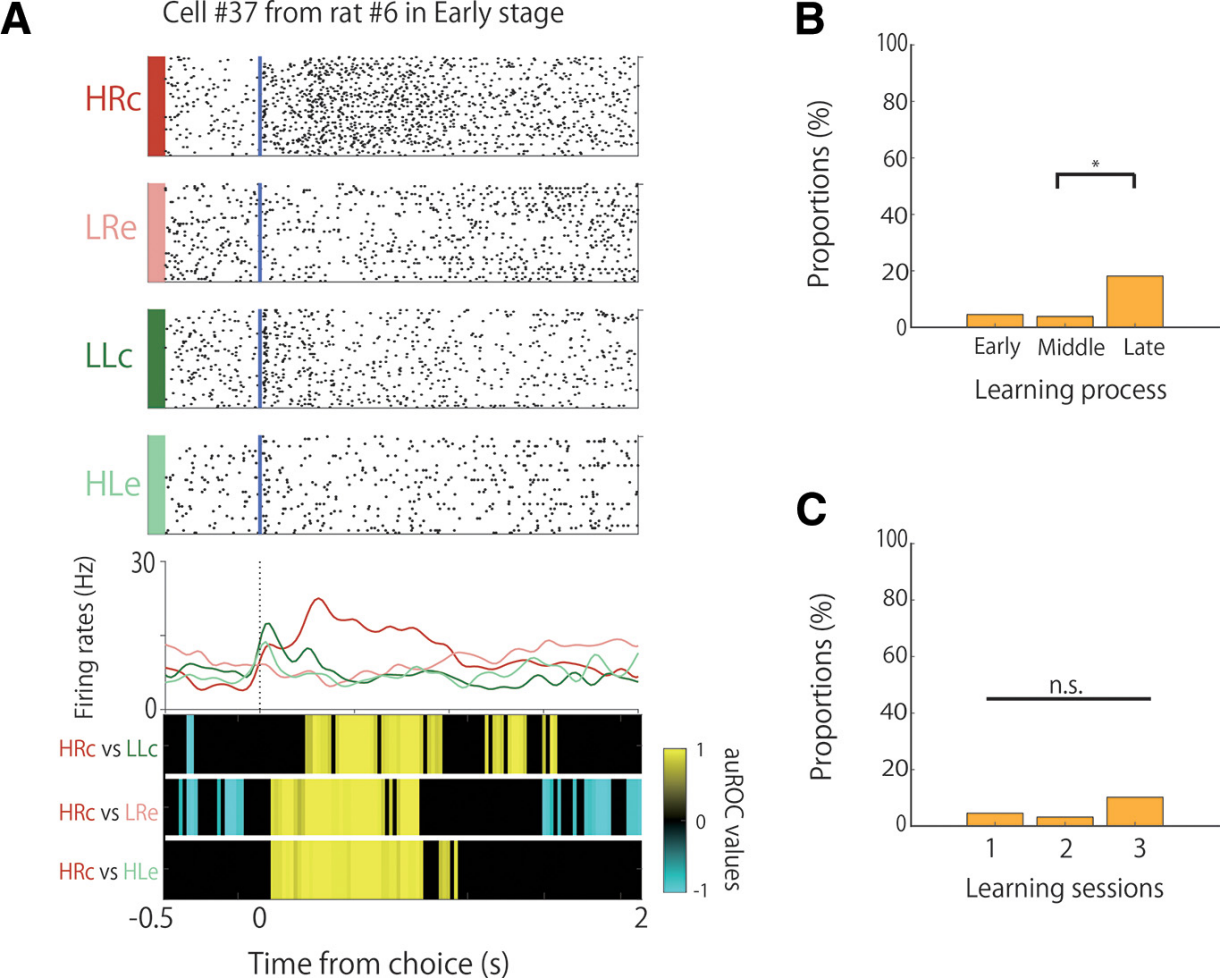
Proportion of reward direction-selective cells during learning. ***A*** An example of the firing patterns of reward direction-selective cells, with firing increases in the HRc trials presented. The data from 0.5 s before to 2 s after the choice responses are shown. Top panels, Raster plots for each task condition. Blue lines indicate the choice response times. Middle panels, PSTHs of each task condition. Dashed lines indicate the choice response times. Bottom panels, auROC values of the task conditions for defining reward direction-selective cells. The color scale represents the auROC values. Nonsignificant auROC values close to 0 are expressed in black. ***B***, Comparison of the proportion of reward direction-selective cells among the learning stages. ***C***, Comparison of the proportion of reward direction-selective cells among the learning sessions. n.s.; not significant; *p <* *0.05.

Reward direction-selective cells modulated their firing rates after the rats chose either of the correct ports (left or right). These cells consist of right-choice reward-selective cells and left-choice reward-selective cells. We defined right-choice reward-selective cells as follows: (1) auROC values of HRc versus LLc that were aligned by choice response time and were significant for five bins in a row from the time of choice to 3 s after the response; (2) auROC values of HRc versus LRe that were aligned by choice response time and were significant for the same periods as in 1; and (3) auROC values of HRc versus HLe that were aligned by choice response time and were significant for the same periods as in 1 (Fig. 5*A*).

We defined left-choice reward-selective cells as follows: (1) auROC values of LLc versus HRc that were aligned by choice response time and were significant for five bins in a row from the time of choice to 3 s after the response; (2) auROC values of LLc versus LRe that were aligned by the choice response time and were significant for the same periods as in 1; and (3) auROC values of LLc versus HLe that were aligned by choice response time and were significant for the same periods as in 1.

We detected a few neurons that responded to two or three events (frequency, choice direction, and reward direction), and each conjunctive cell was counted twice or thrice for each of the three response types (see Table 3).

#### Quantifying the degree of selectivity

We computed a selectivity index to evaluate the degree of selectivity, which was calculated as (*H* – *L*)/(*H* + *L*).” Here, *H* and *L* are the average firing rates of the HRc and LLc trials in the 100 ms around the selectivity peak, respectively.

### Statistical analysis

#### Behavioral performance

One-way ANOVA and multiple-comparison tests were used to compare differences in task performance.

#### Detecting task-related cells

We used auROC analysis and permutation tests because it is usually difficult to predict the type (normal or non-normal) of the distribution of neuronal firing rates and populations of task-related neurons (Sakurai, 1994, 1996). Therefore, our past and recent studies (Sakurai, 1994, 1996; Shiotani et al., 2020; Tanisumi et al., 2021) used distribution-free nonparametric statistics (Siegel, 1956) or ROC analyses (Green and Swets, 1966) rather than parametric analyses (e.g., ANOVA) to analyze neuronal firing rates statistically and to define task-related activity. auROC analysis has an advantage in that we can compare differences between conditions quantitatively using auROC values, whereas we cannot compare them quantitatively with *p*-values in ANOVA.

#### Proportions of task-related cells

The χ^2^ test was used for group effects, and Fisher’s exact test was used to detect a significant difference between the two conditions. The tallies from Fisher’s exact test were corrected using the Holm correction.

#### Strength of selectivity

Selectivity index comparisons between two conditions and among three conditions were analyzed using Wilcoxon rank-sum and Kruskal–Wallis tests, respectively.

## Results

### Learning of the associative memory task and recording of neural activity from A1

Six rats were trained in the associative memory task with frequencies of 3 and 1 kHz as pretraining. The electrodes were implanted when the rats completed the pretraining. A week after the surgery for electrode implantation, the rats began to learn the task at frequencies of 10 and 6 kHz (learning session). The rats performed the task in three to five sessions (i.e., 3–5 d). Table 1 shows the percentage of correct responses from each animal during each session. Figure 1*C* shows examples of the task performance of the following three rats: one was a fast learner (rat 1) and the others were slow learners (rats 3 and 6) that showed different learning curves. We divided the learning sessions into three stages (Fig. 1*C*). The first session was defined as the “early stage” (mean behavioral accuracy = 59.80%, *n* = 6), the last session as the “late stage” (mean behavioral accuracy = 84.41%, *n* = 6), and the sessions before the last session as the “middle stage” (mean behavioral accuracy = 74.49%, *n* = 6). The middle stage could be the second, third, or fourth session, and the last stage could be the third, fourth, or fifth session according to the performance of each animal. The behavioral accuracies of these three stages differed significantly (Fig. 1*D*; one-way ANOVA: *F*_(2,15)_ = 32.18, *p *=* *3.75 × 10^−6^; Tukey’s HSD: early < middle, *p *=* *6.98 × 10^−4^; middle < late, *p *=* *0.02; and early < late, *p *=* *2.56 × 10^−6^). The performance of the rats gradually increased during the first, second, and last stages (Fig. 1*C*). This gradual increase became apparent when the total number of learning sessions was divided into three stages according to the task performance of each animal.

**Table 1:** Task performance (percentage correct) of each rat in each session

	Session 1	Session 2	Session 3	Session 4	Session 5
Rat 1	58.1	65.4	82.3		
Rat 2	70.7	78.2	80.4		
Rat 3	65.1	76.7	71.3	72.6	84.7
Rat 4	49.7	59.5	53.2	77.1	84.2
Rat 5	59.9	78.2	86.8		
Rat 6	55.3	59.0	64.0	75.4	88.0

We also compared the accuracies of the first (early stage), second (mean behavioral accuracy = 69.54%, *n* = 6), and third sessions (mean behavioral accuracy = 72.90%, *n* = 6), but the differences were not significant (Fig. 1*E*; one-way ANOVA: *F*_(2,15)_ = 2.83, *p *=* *0.09). We compared the performances of these sessions to reveal that the differences in neuronal activity depend on increases in task performance (learning) and session numbers (trials). Although task performance seemed to gradually increase during the first, second, and third sessions (Fig. 1*E*), the increase was not statistically significant because of the high variability in each session. However, as described above, the increase in task performance during the early, middle, and late stages was highly significant (Fig. 1*D*).

We recorded the spiking activity of A1 cells of the rats when they were learning the task (Fig. 1*F*, Table 2). We recorded 66, 52, and 44 neurons in the early, middle, and late stages, respectively, and 66, 63, and 49 neurons in the first (early), second, and third sessions, respectively. The tetrodes were not moved, and the constant-spike waveforms of the individual neurons were confirmed within a single session. The spike waveforms were also stable in repeated recordings on successive days (Fig. 1*G*). The stability was quantitatively judged to be reliable when the means of the spike energies for each spike cluster were within ±2 SDs on different days. The waveform consistency also indicates consistency among the neuron types (principal neurons vs interneurons) throughout the learning stages, as principal neurons and interneurons have different spike waveforms. These results suggested that the recordings were most likely from the same cell population. However, occasionally, some neurons disappeared or new neurons appeared between recording sessions (Li et al., 2017), resulting in a variation in the number of recorded neurons among the three stages of learning.

**Table 2:** Number of neurons recorded from each rat in each session

	Session 1	Session 2	Session 3	Session 4	Session 5
Rat 1	16	13	7		
Rat 2	5	7	8		
Rat 3	4	4	2	2	2
Rat 4	14	16	10	10	10
Rat 5	8	8	8		
Rat 6	19	15	14	12	9

### Frequency-selective activity of A1 neurons

We observed frequency-selective cells that increased or decreased their firing rates in response to tone differences (high or low), regardless of whether the choice was left or right, and correct or incorrect (Fig. 2*A*). These frequency-selective cells were observed in each session, but their proportions differed significantly among learning stages (Fig. 2*B*; χ^2^ test, χ^2^ (2) = 16.38, *p *<* *0.01). We observed that 21.2% (14 of 66) of the neurons had frequency selectivity in the early stage, and the proportion of the neurons significantly increased to 59.1% (26 of 44 neurons) in the late stage (Fisher’s exact test with Holm correction; early < late, *p *=* *9.43 × 10^−5^). However, frequency selectivity was observed only in 22% (14 of 63) and 34% (17 of 49) of the neurons in the second and third sessions, respectively, without significant differences among the first, second, and third sessions (Fig. 2*C*; χ^2^ test, χ^2^ (2) = 3.18, n.s.). We did not perform pairwise comparisons of the firing rates between sessions because of the small difference in the number of recorded cells in the sessions.

To assess the difference in the strength of selectivity among the learning stages, we calculated the degree of selectivity of each of the frequency-selective cells based on their firing rates in the high-tone and low-tone trials. There were no significant differences among the learning stages (Fig. 2*D*; Kruskal–Wallis test, *p *=* *0.17) or among the first, second, and third sessions (Fig. 2*E*; Kruskal–Wallis test, *p *=* *0.21).

### Frequency-selective activity engagement in the task

To investigate whether the frequency selectivity of A1 cells was engaged in the task, we compared the firing activity between the associative memory task (27 cells) and the passive tone condition (32 cells) in three of six rats. The tetrodes were not moved, and the recorded cells were from the same cell populations as those used in the task training. In the passive tone condition, 25% (8 of 32) of the neurons showed sound-evoked firing that was modulated by tone presentation, regardless of the tone frequency (Fig. 3*A*). This type of frequency-insensitive firing was not observed in the associative memory task (Fig. 3*B*). Moreover, 44% (12 of 27) and 19% (6 of 32) of the neurons were frequency-selective cells (e.g., Fig. 2) in the task and passive tone conditions, respectively. The proportion of frequency-selective cells was significantly higher in the task than in the passive tone condition (Fig. 3*C*; Fisher’s exact test, *p *=* *4.75 × 10^−2^). Furthermore, the frequency selectivity of the frequency-selective cells was higher in the task than in the passive tone condition (Fig. 3*D*; Wilcoxon rank-sum test, *p *=* *7.54 × 10^−4^).

### Choice direction-selective activity of A1 neurons

We observed choice direction-selective cells during the associative memory task. These cells modulated their firing rates in response to the choice direction (left or right), regardless of whether the tone was high or low and whether the choice was correct or incorrect (Fig. 4*A*). The proportions of these cells were 9% (6 of 66), 19% (10 of 52), and 18% (8 of 44) in the early, middle, and late stages, respectively, without a significant difference among the learning stages (Fig. 4*B*; χ^2^ test, χ^2^ (2) = 2.91, n.s.). The proportions of the choice direction-selective cells in the second and third sessions were 12.7% (8 of 63) and 10.2% (5 of 49), respectively, but there were no significant differences among them (Fig. 4*C*; χ^2^ test, χ^2^ (2) = 0.46, n.s.). We then compared the strength of the choice-direction selectivity, but there were no significant differences among the learning stages (Fig. 4*D*; Kruskal–Wallis test, *p *=* *0.42) or the three sessions (Fig. 4*E*; Kruskal–Wallis test, *p *=* *0.54).

We also observed several choice-direction cells that exhibited frequency selectivity (Table 3). The proportions of these conjunctive cells were 1.5% (1 of 66), 3.8% (2 of 52), and 9.1% (4 of 44) in the early, middle, and late stages, respectively.

**Table 3:** Numbers of task-related cells and conjunctive cells in each learning stage

	Early	Middle	Late
Frequency-selective cells	12	18	18
Choice-direction cells	4	7	1
Reward-direction cells	1	0	1
Frequency- and choice-direction cells	1	2	4
Frequency- and reward-direction cells	1	1	4
Choice- and reward-direction cells	1	1	3

### Reward direction-selective activity of A1 neurons

Some neurons in A1 showed reward direction selectivity. Reward direction-selective cells modulated their firing rates after the rats chose either of the correct ports (left or right; Fig. 5*A*). Therefore, the reward-direction cells consist of both right- and left-choice reward cells.

As shown in Figure 5*A*, there were distinct effects in the immediate phasic response following reward delivery after choosing the left port (LLc). However, there was no significant difference in the phasic response between LLc and HRc, as shown by the auROC values at the bottom of Figure 5*A*. Therefore, the phasic response of this neuron did not exhibit directional selectivity. The auROC values also showed that the prolonged responses that followed the reward delivery after choosing the right port (HRc) contributed to the directional selectivity of this neuron.

Concerning the dichotomy between the early (phasic) and late (prolonged) responses, we analyzed the activity of each reward-direction cell in the phasic and prolonged periods separately. We observed that 10% of the reward-direction cells (2 of 20) showed both phasic and sensory responses after the reward buzzer and prolonged responses corresponding to reward and choice direction. The other reward-direction cells showed only a prolonged response corresponding to the reward and choice directions.

The proportion of these cells differed significantly among the learning stages (Fig. 5*B*; χ^2^ test, χ^2^ (2) = 8.46, *p *<* *0.025). The proportions of these cells were 4.5% (3 of 66) and 3.8% (2 of 52) in the early and middle stages, respectively, but they showed a significant increase to 18% (8 of 44) in the late stage (Fisher’s exact test with Holm correction; middle < late, *p *=* *0.04). However, in the second and third sessions, the proportions of these cells were 3.2% (2 of 63) and 10.2% (5 of 49), respectively, without significant differences among the three sessions (Fig. 5*C*; χ^2^ test, χ^2^ (2) = 2.80, n.s.).

We observed a few reward-direction cells that exhibited frequency selectivity (Table 3). The proportions of these conjunctive cells were 1.5% (1 of 66), 1.9% (1 of 52), and 9.1% (4 of 44) in the early, middle, and late stages, respectively. We also observed a few reward-direction cells that exhibited choice-direction selectivity. The proportions of these conjunctive cells were 1.5% (1 of 66), 1.9% (1 of 52), and 6.8% (3 of 44) in the early, middle, and late stages, respectively.

## Discussion

In this study, we examined neuronal activity in A1 during the entire process of learning an auditory associative memory task. We found that A1 neurons showed selectivity for tone frequencies, choice directions, and reward directions. The proportion of frequency-selective and reward-direction cells increased during task acquisition. Therefore, A1 neurons showed learning-dependent modulation of activity when associations between auditory tones and behavioral responses were gradually induced by learning.

The proportion of frequency-selective cells was learning dependent and increased significantly in the late stages of the learning process (Fig. 2*B*). This increase in the proportion was not caused by a simple increase in sessions, but by a significant increase in correct performance because of learning. This conclusion was derived because the proportion of cells did not differ significantly among the first, second, and third sessions, wherein the correct performance on the task did not increase (Fig. 2*C*). Furthermore, the selectivity did not appear to be because of the difference between the correct and incorrect trials. This is because the auROC values of the HRc versus HLe and LLc versus LRe conditions were not significant in high-frequency-selective cells and low-frequency-selective cells, respectively. These comparisons indicated that the activity of the frequency-selective cells was modulated only by frequency and not by correct or erroneous trials. The strength of selectivity in these cells, however, did not differ significantly between the learning stages and sessions (Fig. 2*D*,*E*). The strength of the selectivity had to increase for each cell to become frequency selective. However, learning dependency was related to the increase in the number of frequency-selective cells and not to the increase in the strength of each selective cell. These results suggest that tone frequency selectivity in A1 increases during the learning process by increasing the number of frequency-selective cells but not by increasing the strength of the frequency selectivity of each cell. This modulation of proportions might be related to plastic changes in the tonotopic map related to the learning task, as the tetrodes were not moved throughout the successive days of training and the recording sites were not changed. The proportion of frequency-selective cells was also significantly different between the task and passive tone conditions (Fig. 3*C*), and the strength of the selectivity was significantly higher in the former than in the latter (Fig. 3*D*). Furthermore, some frequency-selective cells showed phasic modulation in brief periods following tone presentation, whereas others showed tonic modulation during tone presentation. These results suggest that the frequency selectivity of A1 neurons emerges strongly when the tone is engaged in a task with choices and rewards.

Some previous studies reported that the representation area of tone frequency in A1 increased with learning (Recanzone et al., 1993; Polley et al., 2006), and that enlargement occurred very quickly in several trials (Edeline and Weinberger, 1993; Fritz et al., 2003, 2005). However, in our results, the number of frequency-selective cells increased over a longer time span of several days. This might be because of the differences in the demands and difficulties of the tasks between previous studies and this study. In other words, the conditions may have been simpler in the former than in the latter, which could have been acquired slowly. This notion is supported by previous studies reporting that the sound-evoked activity of A1 neurons is modulated by task engagement (Atiani et al., 2009; De Franceschi and Barkat, 2021) and task structures (David et al., 2012).

A1 neurons also showed choice-direction selectivity, and the proportion of these cells did not differ significantly among learning stages (Fig. 4*B*). These cells might not be affected by the different acoustic properties of the different parts of the experimental chamber, as the distance between the right and left ports was only 70 mm and the distance from the loudspeaker was 550 mm for both ports. Furthermore, the selectivity of the choice-direction cells was not affected by the difference between the correct and erroneous trials. This is because the auROC values of the HRc versus LRe and LLc versus HLe conditions were not significant for right choice-selective and left choice-selective cells, respectively. This indicates that A1 neurons represent tone signal and behavioral choice information, and these representations are stable throughout the learning process.

The activity of A1 neurons in response to behavioral movements has been reported in previous studies (Nelson et al., 2013; Rummell et al., 2016). In addition, Guo et al. (2019) reported that A1 and posterior striatum neurons show choice-direction selectivity based on reward expectations. Choice-related activity has also been found in other primary sensory cortices, such as the visual cortex (Stringer et al., 2021) and olfactory cortex (Miura et al., 2012; Poo et al., 2022). In our previous study, choice direction-selective cells were also found in the hippocampal CA1 region in the same associative memory task, and the proportion of these cells did not differ significantly among the learning stages (Takamiya et al., 2021). These responses in previous studies were not necessarily associated with the formation of associations that led to the consolidation of associative memory. Therefore, it could be suggested that choice directions in associative learning are represented in several brain regions, including in the sensory cortices and hippocampus, and such representations have stable functions throughout the learning process.

We found that some A1 neurons showed reward-direction selectivity, and the proportion of reward-direction selectivity in the late stage of the learning process was significantly higher than that in the early stage (Fig. 5*B*). This suggests that A1 neurons represent reward directions as well as tone frequencies and choice directions, and the proportion of these cells increases when the rats learned the associative memory task. The increases in the reward-direction cells did not depend on the number of sessions, but on the accuracy of the task. This conclusion was drawn because the proportion of these cells did not significantly differ among the first, second, and third sessions, wherein there was no improvement in accuracy (Fig. 5*C*). The activity of the reward-direction cells was not affected by the buzzer sounds related to reward delivery or by sounds from the food delivery system or motor aspects of reward consumption, as these sounds were present in both the right-choice and left-choice reward trials. If these sounds from rewarded trials affected neuronal activity, the neurons should have shown the same firing rate modulations in both the right-choice and left-choice reward trials. The activity of these cells was also unaffected by an increase in the number of rewarded trials.

A few reward-directed cells showed both phasic and prolonged responses after reward delivery (Fig. 5*A*). The prolonged responses could correspond to reward intake, as they appeared from 0.5 s after the choice and reward, and continued for a short time. However, it should be noted that this prolonged activity was dependent on the choice direction and did not simply reflect the reward intake itself. In contrast, the phasic response corresponded to the sensory input of the reward buzzer and was not dependent on the reward direction. The phasic response could correspond to input from primary sensory pathways, whereas activity during the remaining prolonged time could correspond to signals from other cortical and/or other areas.

Our previous study (Takamiya et al., 2021) suggests that the activity of reward-direction cells in the hippocampal CA1 reflects positive feedback of the correct port choice to form an association between auditory stimuli and port directions. In that study, the proportion of reward-directed cells in the hippocampal CA1 increased in the middle stage and decreased in the late stage of the learning process. According to the two-stage model (Buzsáki, 1996, 2015), the hippocampus could be called the “fast learner,” as it rapidly encodes information via changes in synaptic strength during behavioral acquisition. The information is repeatedly replayed during slow-wave sleep and is gradually transferred to the neocortex, resulting in a hippocampal–neocortical interaction that could be referred to as a “slow-learning” interaction. Thus, the hippocampus and auditory cortex interact with each other, and the interaction between them plays an important role in memory formation and leads to consolidation (Rothschild et al., 2017). The activity of the reward-direction cells in the A1 region may reflect positive feedback, thereby forming and stabilizing auditory associative memories. The actual interaction between hippocampal and A1 neurons during the formation of auditory associative memory should be examined in the near future.

## Synthesis

Reviewing Editor: David Schoppik, New York University - Langone Medical Center Decisions are customarily a result of the Reviewing Editor and the peer reviewers coming together and discussing their recommendations until a consensus is reached. When revisions are invited, a fact-based synthesis statement explaining their decision and outlining what is needed to prepare a revision will be listed below. The following reviewer(s) agreed to reveal their identity: John Middlebrooks.

Both reviewers agreed this was an interesting study, but expressed concern that the statistical analyses necessary to support the conclusions were incomplete and potential confounds were not adequately considered. The reviewers took considerable effort to go line-by-line to point out where the manuscript could be improved and where they struggled so I will include a lightly edited consolidation of their points below. We all believe that the majority of these can be addressed with textual changes that we think will make the manuscript stronger.

Major points:

Line 8: It would be better to say “tone frequencies” than “tone pitches”. Frequency is the actual physical parameter that was varied, whereas pitch is the assumed percept. Also, in complex sounds (unlike the present study), there is a non-trivial relationship between frequency content and pitch.

Lines 41-43: This is confusing. It says that A1 neurons are intrinsically sensitive to a specific tone pitch, yet it then says that the neurons become tuned to frequency of a conditioned tone. If the pitch sensitivity is intrinsic, do you mean that the tuning becomes sharper or that the proportion of neurons tuned to the conditioned tone increases? Also, “pitch” is really the wrong word here. A1 neurons are tuned to pure-tone frequencies. A complex of high-frequency tones might elicit the same pitch as a lower-frequency pure tone, but the high-frequency complex would activate a different population of A1 neurons than would the lower-frequency tone.

Line 56: What do you mean by “a longer time”? Days? Weeks?

Line 89, The task protocol could be made more clear. It seems that some animals learned high tone-left reward associations and others learned high tone-left reward associations. If so, was the sampling homogeneous? Was the “random” selection of high vs low tone truly random (permitting for example, runs of 6 successive high tone trials) or (preferably) was it pseudo-random, not permitting long runs, rewarding alternation behavior, and with equivalent numbers of rewarded left and right trials. If the former, were there any long runs? This could be a potential confound if, for example, a cell’s choice direction was right, and this animal had a high-tone/right reward association, and then, in the final stage of learning (with mostly right choices) these cells would also be considered reward-direction cells for right rewards. (It would also have been preferable for the experimental design to add a variable delay between the nosepoke responses and visual and auditory signals provided in the behavioral paradigm for rewards and for errors to better distinguish choice direction and reward direction responses.)

Is Figure 5A real data, or a hypothetical “representation”? Since the numbers of rewarded and error trials are equal, this would be at an early or middle stage of learning where there were very few reward direction cells. If this is real data, please state the training stage and day.

Associated with this, on lines 269-270, “modulated their firing rates after receiving a reward for choosing the correct port (left or right)”. This phrase could be made more clear. Furthermore, the example/‘representation’ is not convincing when comparing the auROC yellow zones. There seems to be a prolonged response for reward right, but not reward left. There is also a possible partial confound since error trials have a buzzer sound (are they buzzer-right or buzzer-left responses?). And rewarded trials would have possible confounds from the sound of the food delivery system and motor aspects of reward consumption (and chewing makes sounds and vibrations that could affect the inner ear). These could be influential since these responses are measured over a 3 s period after the reward delivery. Thus the responses could be associated with sensory and/or motor events, and perhaps not with learning the tone-direction association. The greater proportion of rewarded trials with learning could be associated with the detection of greater proportions of rewarded responses at the last stage, creating a larger (and hence lower variance) sample than of unrewarded responses.

Lines 155-159: It is not clear what the difference is between a “sliding ROC analysis” (line 155) and an “auROC analysis”. “auROC” is not a term that is used in the major signal-detection sources (e.g., Green and Swets; Macmillan and Creelman). The mathematical expression is given in the Shiotani et al. citation that you give, but even that doesn’t define the acronym. I guess that auROC is “area under the receiver operating characteristic curve", but I shouldn’t have to guess. Also, given this uncommon usage, you should tell us in this manuscript that auROC ranges from -1 to +1 depending on whether the spikes favor one or the other value of the parameter. (In a more typical usage, one would just have the area under the ROC curve ranging from 0 to 1, with 0.5 indicating chance performance.)

Line 164: It would be better to say “frequency-selective cells” than “tone-selective cells”. That is because, in line 169 and Fig. 3, you define “sound-evoked cells” that respond to either of the tone frequencies. The only sounds in this study are tones, however, so a reader might confuse sound-evoked cells with tone-selective cells. I don’t understand these definitions. In (1), you say comparing firing rates of “HRc (LLc) and the LLc and LRe (HRc and HLe) trials”. I originally took that to mean that you compared HRc versus LLC or compared LLC and LRe versus HRc and HLe). Now I think that you mean you compared HRc versus LLc and LRe or compared LLc versus HRc and HLe. The latter explanation is the only way that (2) makes sense. Please clarify this.

All the raster plots in Figs. 2A, 3A, 4A, and 5A need time scales. Please tell us in the presentation of Fig. 2 in the main text that the yellow and blue lines correspond to tone onset and choice response. Nevertheless, how can there be a fixed time delay between tone onset and the animal’s choice? There is the same problem in Fig. 4A. It seems as if the raster plots here are aligned by the choice time (the blue line), but then how can the tone onset be a constant that can be denoted by the orange line?

Line 179: Please indicate here in the main text that the raster in Fig. 5A is aligned by choice response time, not stimulus onset.

line 201: I think that you mean “three stages” rather than “three groups”. Maybe this means groups of sessions, but everywhere else you use “stages”.

Throughout the manuscript three or four significant digits are used to report results. In light of the precision of the data, the results should also only contain two significant digits (e.g., line 206).

Line 210, and elsewhere. It should be made more clear why the comparisons of 1st, 2nd and 3rd sessions is of interest, since there were no behavioral differences in these sessions.

Line 212, please provide rigorous quantitative criteria for confirmation of spike waveform consistency. Does this concern stability of waveforms within single sessions, or repeated recordings of the same cell on successive days? Is it possible that the same cell was recorded on successive days? If so the tallies of proportions of responses should be corrected since they could be inflated by repeated measures of the same cell.

Line 214-215, “some neurons disappeared, or new neurons appeared during the recording” Did this happen within the recording sessions? This could introduce sampling problems. If between sessions, then this is related to the previous question concerning line 212.

Lines 219-221 (and below for other response types), “tone selective cells that modulated their firing rates in response to tone differences (high or low), regardless of whether the choice was left or right and correct or incorrect.” Does this mean that none of the neurons counted had responses for more than one of the events (tone, choice-direction, reward-direction)? This must be clarified and quantified it there were multiple responses for a given neuron. Line 224 is ambiguous about this: are these numbers for cases when tone-selectivity was the only response of these cells, or could some cells be counted twice for the respective tallies for the three response types?

Line 218: Again, it would be better to say “Frequency selective” here, especially because the heading in line 238 says “tone selective” when the following 6 lines refer to responses to any sound, irrespective of frequency.

Line 224 “which increased to 40.4%”. If it wasn’t significant, then the sentence cannot say that it increased.

Lines 242-243, “the recorded cells were from the same cell populations as those of the task training” This conclusion requires further justification, in particular because neuron types (e.g., principal neurons vs interneurons) are not described. If the same cells were recorded in two sessions, pair-wise comparisons would seem appropriate.

Line 244, “that responded to tone onset” What is the response delay and duration criterion distinguishing responses to tone onset (which suggests a phasic response) vs tonic responses during the more prolonged tone period as described in the Methods section?

Line 245: It would be helpful to say “This type of frequency-insensitive firing” to distinguish from the frequency selective cells in the next sentence.

Line 250, please clarify and quantify the measures of “strength of tone selectivity”. The methods section says that this was computed over 100 ms intervals. The plots of auROC values over time show quite variable responses, positive and negative and it is not clear how this would be computed to give a single value for these periods. What is the significance criterion value for auROC values? What is the reasoning behind taking the absolute selectivity index, and how was this done? Would this average the magnitudes of positive and negative values (significant and not significant) throughout the respective periods?

Are the tone-selective results related to:

1) different positions of recording sites on tonotopic map?

2) plastic changes in the tonotopic map related to learning the task?

With such small samples, estimates of statistical strength would help establish the reliability of results.

Line 285-287, “Therefore, the A1 neurons show learning-dependent modulation of

activity when associations between auditory tones and behavioral response are gradually made by learning.” Alternative interpretations: change of tonotopic organization with training, and reward delivery/chewing noise/error buzzer responses.

Line 290, “the proportion did not depend on the number of sessions” This is ambiguously stated and could be interpreted as related to whether the animal had 3, 4 or 5 sessions.

"accuracy” can alternatively be interpreted in terms engagement of the animal to the task and cues (as mentioned elsewhere) which could, in turn, be associated with the degree of plastic changes in the tonotopic responses.

Lines 295-297: This point would be easier to see if you were to indicate the number of cells in each box in Figs. 2D, 2E, 3D, 4D, and 4E.

Lines 296-7, “not by increasing the strength of the tone selectivity of each cell” Didn’t the strength have to increase for more cells to become significant?

Line 314, Could choice-direction responses be related to different acoustical properties of different parts of the experimental chamber?

Line 317-319, “The activities of the A1 neurons that responded to behavioral movements have been reported in some previous studies”. Then these responses are not necessarily associated with “the formation of associations leading to consolidation of associative memory”.

Line 523: There are no blue lines in these rasters. Please clarify that, I think, these rasters are from the passive condition. Please label panels B and C to indicate that B shows proportions of “sound-evoked” frequency-insensitive, whereas C shows frequency-selective cells. Also, indicate in panel D that you are plotting the selectivity index for frequency selectivity. Otherwise, the reader would look at 3D and assume that 3D plots selectivity for sound versus no sound.

Minor comments:

Although the use of the word “we” is acceptable, its appearance in three consecutive sentences in the abstract appears stilted.

Line 51, replace the word “obvious”. Also rephrase “associative memory that forms associations” to avoid word repetition.

Line 56, longer than what?

Lines 78-79, an LED light is an infrared sensor? Contradictory.

Line 146, please provide the link for MClust, rather than just a name, facilitating replication of results.

Line 148, please quantify the criterion for “reliable refractory periods"

Line 200, presumably the middle stage does not include the first session, but this formulation does not make this clear.

Line 504, “cells” How many cells are shown here?

Lines 504-505,"firing increments” Please restate more clearly and precisely (when do increments occur? Are all responses increments, or are some reductions of tonic firing?) Do all TS cells show effect only in brief period after tone? Why then is such a long period used for characterizing this category?

Statistical data are repeated both in the text and in Figure Legends, sometimes incompletely. This could be consolidated.
